# The Role of Iron in Atherosclerosis in Apolipoprotein E Deficient Mice

**DOI:** 10.3389/fcvm.2022.857933

**Published:** 2022-05-20

**Authors:** Juan Ma, Hui-Min Ma, Meng-Qi Shen, Yuan Yuan Wang, Yu-Xin Bao, Yong Liu, Ya Ke, Zhong-Ming Qian

**Affiliations:** ^1^Institute of Translational and Precision Medicine, Nantong University, Nantong, China; ^2^Laboratory of Neuropharmacology, Fudan University School of Pharmacy, Shanghai, China; ^3^National Clinical Research Center for Aging and Medicine, Huashan Hospital, Fudan University, Shanghai, China; ^4^Research Center for Medicine and Biology, Zunyi Medical University, Zunyi, China; ^5^Department of Pain and Rehabilitation, The Second Affiliated Hospital, The Army Medical University, Chongqing, China; ^6^School of Biomedical Sciences and Gerald Choa Neuroscience Center, Faculty of Medicine, The Chinese University of Hong Kong, Sha Tin, Hong Kong SAR, China

**Keywords:** atherosclerosis, apolipoprotein E (ApoE), iron transport and regulation, reactive oxygen species (ROS), iron-mediated atherosclerosis

## Abstract

The role of iron in atherosclerosis is still a controversial and unsolved issue. Here, we investigated serum iron, expression of iron regulatory, transport and storage proteins, pro-inflammatory chemokines and cytokines in ApoE^–/–^ mice. We demonstrated that ApoE^–/–^ induced atherosclerosis and an increase in iron contents, expression of transferrin receptor 1 (TfR1), iron regulatory proteins (IRPs), heme oxygenase 1 (HO1), cellular adhesion molecules and pro-inflammatory cytokines, production of reactive oxygen species (ROS), and a reduction in expression of superoxide dismutase and glutathione peroxidase enzyme in aortic tissues. All of these changes induced by ApoE deficiency could be significantly abolished by deferoxamine. The data showed that the increased iron in aortic tissues was mainly due to the increased iron uptake *via* IRP/TfR1 upregulation. These findings plus a brief analysis of the controversial results reported previously showed that ApoE deficiency-induced atherosclerosis is partly mediated by the increased iron in aortic tissues.

## Introduction

Atherosclerosis, a leading cause of death worldwide, is an almost inevitable consequence of ageing, food preferences, lack of exercise and other aspects of the lifestyle found in many countries (1). The pathology of atherosclerosis is characterized by the accumulation of lipids and inflammatory cells, including macrophages, in blood vessel walls and the thickening of the arterial intimae and formation of atherosclerotic plaque (2–4). Endothelial activation, which is characterized by up-regulation of cellular adhesion molecules and pro-inflammatory chemokines and cytokines, and consequent monocyte recruitment to the arterial intimae are etiologic factors in atherosclerosis (5).

Abnormally increased iron in tissues has been implicated as a major generator of reactive oxygen species (ROS) which are capable of damaging biological molecules such as DNA, lipids, carbohydrates, and proteins (6). During past decades, iron has long been suspected to promote the development of atherosclerosis because of its role in the formation of ROS (7–12). However, up to now it is still a controversial and unsolved question of whether iron is involved in the development of atherosclerosis (1, 4, 13–17). The role of iron in the pathogenesis of atherosclerosis is still not completely understood (11, 12, 15).

Apolipoprotein E (ApoE) is a physiological ligand for the low density lipoprotein receptor (LDLR), acting as an important modulator of lipoprotein metabolism (18). ApoE-deficient (ApoE–/–) mice have been widely used in preclinical atherosclerosis studies because of the propensity to spontaneously develop atherosclerotic lesions with features similar to those seen in humans (19). It has also been demonstrated that there is a progressive accumulation of iron with age in atherosclerotic lesions and tissue sections of the heart and liver (20), and in liver and spleen tissues in ApoE deficient mice (21). These findings led us to hypothesize that ApoE might have an inhibitory effect on iron contents in the cells or tissues by downregulating iron uptake proteins and/or up-regulating iron release protein under physiological conditions. In the absence or reduced expression of ApoE, their physiological inhibition of iron content in cells will be relieved or partially relieved, and iron in cells and tissues will be abnormally increased.

To test this hypothesis, we investigated the changes in the serum iron, expression of iron regulatory, transport and storage proteins, cellular adhesion molecules and pro-inflammatory chemokines and cytokines in ApoE–/– mice treated with deferoxamine (DFO) in the present study. We also discussed the possible reasons why patients with hereditary hemochromatosis do not show any increased incidence of atherosclerosis and analyzed some conflicting results from several animal studies. Based on our findings and the discussion and analysis, we concluded that ApoE deficiency induced atherosclerosis is partly mediated by the increased iron in aortic tissues in mice.

## Materials and Methods

### Materials

Unless otherwise stated, all chemicals, including mouse monoclonal anti-β-actin and oxidized-low density lipoprotein (ox-LDL) were obtained from the Sigma Chemical Company, St. Louis, MO, United States. Mouse anti-human transferrin receptor 1 (TfR1), Alexa Fluor 488 goat anti-rabbit IgG, TRIzol reagent, RPMI-1640 medium and fetal bovine serum (FBS) were purchased from Invitrogen Life Technologies, Carlsbad, CA, United States; rabbit polyclonal anti-mouse ferroportin 1 (Fpn1) from Novus Biologicals, Littleton, CO, United States; rabbit polyclonal anti-ferritin light chain (FTL) from Proteintech, Chicago, IL, United States; rabbit polyclonal anti-ferritin heavy chain (FTH) from Bioworld Technology Inc., Louis Park, MN, United States; rabbit anti-intercellular adhesion molecule-1 (ICAM1), rabbit anti-vascular cell adhesion molecule-1 (VCAM1), rabbit anti-heme oxygenase 1 (HO1), rabbit anti-glutathione peroxidase 4 (Gpx4), rabbit anti-iron regulatory protein 1 (IRP1) and -IRP2 from Abcam, Cambridge, MA, United States; rabbit anti- Nuclear Factor-kB p65 (NF-κB) and rabbit anti-phospho-NF-κB p65 from Cell Signaling Technology Inc., Beverly, MA, United States; goat anti-rabbit or anti-mouse IRDye 800 CW secondary antibody from LI-COR Biosciences, Lincoln, NE, United States. AevertAid First Strand cDNA Synthesis kit and BCA protein assay kit were obtained from Thermo Scientific, Waltham, MA, United States; Faststart Universal SYBR Green Master and LightCycler96 from Roche, Nutley, NJ, United States; and protein RIPA lysis buffer from the Beyotime Institute of Biotechnology, Haimen, JS, China. All solutions were prepared fresh, prior to each assay.

### Animals and Treatments

ApoE–/– and wild-type (WT, age and gender matched C57-BL/6) mice were purchased from Hua Fu Kang biotechnology Co., Ltd., (Beijing, China) and verified by RT-PCR (Supplementary Figure 1). A total of 27 male mice (*n* = 7 in WT and *n* = 20 in ApoE–/–) were used in this study. ApoE–/– mice were treated with DFO (ApoE–/– + DFO mice, *n* = 10) or without DFO (ApoE–/– mice, *n* = 10) respectively. ApoE–/– mice in ApoE–/– + DFO group received daily i.p. injections of 100 mg/kg body weight (b.w.) of DFO in Hanks’ balanced salt solution (HBSS) for 14 days before sacrifice. All mice were sacrificed at 28-weeks-old for the designed measurements. The mice were housed under specific pathogen-free conditions at 22 ± 2°C with a relative humidity of 60–65% and maintained under a 12-h light/12-h dark cycle and laboratory rodent diets for mice (PMI Nutrition International, the Richmond Standard) and distilled water were freely accessible to the animals throughout the experimental period as previously described (22, 23). All animal care and experimental protocols were performed according to the Animal Management Rules of the Ministry of Health of China, and approved by the Animal Ethics Committees of Nantong University (NDFC31271132) and The Chinese University of Hong Kong (GRF14111815).

### Sampling of Blood and Aorta

Animals were fasted for 8-h and then anesthetized with 1% pentobarbital sodium (40 mg/kg body weight, i.p.) and decapitated. Fasted blood samples were collected (24, 25) for subsequent measurements and the entire thoracic aortas (from the branches of the abdominal aorta to the aortic arch) were bluntly isolated (26) for general morphological and immunohistochemical detection, RNA extraction, western blot analysis and Oil Red O staining assay.

### Isolation of Total RNA and Quantitative Real-Time PCR

Total RNA extraction and cDNA preparation were performed using TRIzol reagent and the AevertAid First Strand cDNA synthesis kit respectively, in accordance with the instructions of the manufacturers. Quantitative real-time PCR was carried out using FastStart Universal SYBR Green Master and Light Cycler96. The specific pairs of primers of mouse β-actin, hepcidin, IRP1, IRP2, TfR1, Fpn1, FTL, FTH, ICAM1, VCAM1, CD36 (an important receptor for oxidized lipoproteins), LOX1 (Lectin-like ox-LDL receptor 1), glutathione peroxidase 4 (Gpx4), HO-1, tumor necrosis factor-α (TNFα), interleukin 6 (IL-6), interleukin-1β (IL-1β) and interleukin-10 (IL-10) are listed in Table 1. The CT values of each target gene were normalized to that of β-actin mRNA. Relative gene expression was calculated by the 2-ΔΔCT method (27).

**TABLE 1:** The specific pairs of primers.

Primers	Primer sequence
β-actin forward	5′-AAATCGTGCGTGACATCAAAGA-3′
β-actin reverse	5′-GCCATCTCCTGCTCGAAGTC-3′
m-Hepcidin forward	5′-CTGAGCAGCACCACCTATCTC-3′
m-Hepcidin reverse	5′-TGGCTCTAGGCTATGTTTTGC-3′
Hu-Hepcidin forward	5′-CAGCTGGATGCCCATGTTC-3′
Hu-Hepcidin reverse	5′-CAGCAGCCGCAGCAGAA-3′
m-IRP1 forward	5′- ACAGGCCGCGAGGAAGA-3′
m-IRP1 reverse	5′-GAAACATGCCTACAGCCTGAAGAT-3′
Hu-IRP1 forward	5′-TGCTTCCTCAGGTGATTGGCTACA-3′
Hu-IRP1 forward	5′-TAGCTCGGTCAGCAATGGAGAACT-3′
m-IRP2 forward	5′- GCCATAGCAGGCACAGTGAATA-3′
m-IRP2 reverse	5′- TTTCCTTGCCCGTAGAGTCAGT-3′
Hu-IRP2 forward	5′-GATTTTGCTGCTATGAGGGAGG-3′
Hu-IRP2 reverse	5′-GAGAGAGCTTTCCTGCTTTCTG-3′
m-TfR1 forward	5′-TGGGTCTAAGTCTACAGTGGC-3′
m-TfR1 reverse	5′-AGATACATAGGGCGACAGGAA-3′
Hu-TfR1 forward	5′-CTCAGTTTCCGCCATCTCAGT-3′
Hu-TfR1 forward	5′-GCAGCTCTTGAGATTGTTTGCA-3′
m-Fpn1 forward	5′-TCACCTGGCTACGTCGAAAAT-3′
m-Fpn1 reverse	5′-GCTGGGCTAGTCCTGAGAATAGAC-3′
m-FTL forward	5′-CGGGCCTCCTACACCTACCT-3′
m-FTL reverse	5′-CCCTCCAGAGCCACGTCAT-3′
m-FTH forward	5′- AAGATGGGTGCCCCTGAAG-3′
m-FTH reverse	5′- CCAGGGTGTGCTTGTCAAAGA-3′
m-ICAM1 forward	5′-TACGTGTGCCATGCCTTTAGC-3′
m-ICAM1 reverse	5′-GCCCACAATGACCAGCAGTA-3′
m-VCAM1 forward	5′-GAACCCAAACAGAGGCAGAG -3′
m-VCAM1 reverse	5′-GGTATCCCATCACTTGAGCAG-3′
m-HO1 forward	5′-GCTGGTGATGGCTTCCTTGT-3′
m-HO1 reverse	5′-TTGTTGCGCTCTATCTCCTCTTC-3′
Hu-HO1 forward	5′-CTTCTTCACCTTCCCCAACA-3′
Hu-HO1 reverse	5′-TTCTATCACCCTCTGCCTGA-3′
m-CD36 forward	5′-GAACCACTGCTTTCAAAAACTGG-3′
m-CD36 reverse	5′-TGCTGTTCTTTGCCACGTCA-3′
m-LOX-1 forward	5′-CCTCTACCTCAGTATGCCTCCT-3′
m-LOX-1 reverse	5′-CTTTGCATGTTATTTCTCGGACGA-3′
m-Gpx4 forward	5′-TGCATCGTCACCAACGTGGC-3′
m-Gpx4 reverse	5′-CTTCACCACGCAGCCGTTCT-3′
m-TNFα forward	5′-CATCTTCTCAAAATTCGAGTGACAA-3′
m-TNFα reverse	5′-TGGGAGTAGACAAGGTACAACCC-3′
m-IL-6 forward	5′-GAGGATACCACTCCCAACAGACC-3′
m-IL-6 reverse	5′-AAGTGCATCATCGTTGTTCATACA-3′
m-IL-1βforward	5′-GCAACTGT TCCTGAACTCAACT-3′
m-IL-1βreverse	5′-ATCTTTTGGGGTCCGTCAACT-3′
m-IL-10 forward	5′-GCTCTTACTGACTGGCATGAG-3′
m-IL-10 reverse	5′-CGCAGCTCTAGGAGCATGTG-3′

### Western Blot Analysis

Cells and tissues were washed and homogenized as described previously (28, 29). After centrifugation at 14,000 rpm for 15 min at 4°C, the supernatant was collected and protein content was determined using the BCA protein Assay kit. Proteins were heated for 10 min at 100°C in sample buffer [125 mM Tris/HCl, 2% (w/v) SDS, 5% (v/v) 2-mercaptoethanol, 10% (v/v) glycerol, 0.001% (w/v) bromphenol blue, pH 6.8]. Aliquots of the extract containing about 30 μg of protein were loaded and run on a single track of 12% SDS-PAGE under reducing conditions and were subsequently transferred to a pure nitrocellulose membrane. The blots were blocked and then incubated with primary antibodies (Supplementary Table 1) overnight at 4°C. After being washed, the blots were incubated with goat anti-rabbit or anti-mouse IRDye 800 CW secondary antibody (1:5,000) for 2-h at 37°C. The intensity of the specific bands was detected and analyzed by Odyssey infrared image system (Li-Cor, Lincoln, NE, United States).

### Tissue Iron Measurement

The total iron in the tissues was determined using a graphite furnace atomic absorption spectrophotometer (GFAAS) as previously described (30). In brief, tissues were homogenized in 20 mM HEPES followed by digestion in equal volume of ultra-pure nitric acid and then were measured with a GFAAS (Perkin-Elmer, Analyst 100).

### Serum Iron and Ferritin

Serum iron (SI) and unsaturated iron binding capacity (UIBC) were measured using commercial kits as described previously (31, 32). Total iron binding capacity (TIBC μg/dL = serum iron + UIBC) and transferrin saturation (Tf% = SI/TIBC × 100) were calculated. Serum ferritin levels were determined using an ELECSYS 2010 analyzer (Roche Diagnostics GmbH).

### Plasma Redox Status Assessment

Plasmamalondialdehyde (MDA) was assayed by monitoring the formation of thiobarbituric acid reactive substance formation (33). ROS was determined with fluorometric assay using 2′,7′-dichlorofluorescein. The enzymatic activities of H2O2, glutathione peroxidase (GSH-PX) and superoxide dismutase (SOD) were measured using commercial kits according to the instructions of the manufacturer (Nanjing Jiancheng Institute of Biotechnology, Nanjing, China).

### Determination of 8-Isoprostane

8-isoprostane was determined by using an ELISA kit (ab175819). Tissues and blood samples were processed according to the manufacturer’s instructions. Each ELISA sample was tested in duplicates according to the manual of 8-isoprostane ELISA kit. Absorbance readings at 450 nm were normalized to readings of maximum binding control, and quantified into pg/ml using an 8-isoprostane standard curve (34).

### Oil Red O Staining

After dissection, fresh samples were frozen immediately for frozen section preparation. The aortic arch sections were affixed to microscope slides, air-dried at room temperature for 30 min, and then stained with filtered and fresh Oil Red O (Sigma-Aldrich, St. Louis, MO, United States), as described by Nunnari et al. (35). After being washed with PBS, the slides were viewed using an Olympus BX51 microscope (Olympus).

### Immunohistochemistry, Immunofluorescence and DAB-Enhanced Perls Staining

Formalin-fixed paraffin-embedded tissues were sectioned into 0.5 μm sections and stored at room temperature. Sections were deparaffinized using xylene and then were rehydrated in ethanol before being subjected to heat-activated antigen retrieval in citrate buffer (pH 6). Slides then were blocked for non-specific binding using protein block, and endogenous peroxidase activity was quenched. For immunohistochemistry, slides were stained with rabbit primary antibodies: mouse anti-TfR1 (1:100), rabbit anti-FTL (1:100), rabbit anti-ICAM (1:200) and rabbit anti-ICAM (1:150), and then were developed using the Dako Envision HRP-DAB system. All slides were counterstained with hematoxylin and then were visualized using a standard bright field microscope (36). For immunofluorescence, slices were incubated in blocking solution followed by overnight incubation at 4°C with primary antibodies: rabbit anti-IRP1 (1:100), rabbit anti-IRP2 (1:150), rabbit anti-HO1 (1:200) and rabbit anti-hepcidin (1:50). After washing with PBS, the slides were incubated with Alex Fluor 488, conjugated secondary antibodies for 1-h at 37°C. The nuclei were counterstained with DAPI. The slides with immunofluorescence staining were visualized with a confocal microscope (Carl Zeiss; Heidenheim, Germany) (37, 38). For DAB-enhanced Perls staining, slides were immersed for 1-h in 1% potassium ferricyanide in 0.1 MHCl buffer and then were stained with DAB chromogen substrate. The images from at least four fields each from three mice were examined.

### Statistical Analysis

Statistical analyses were performed using Graphpad Prism 5 (Graphpad software, La Jolla, CA, United States). Data were presented as mean ± SEM. The differences between the means were all determined by one or two-way analyses of variance (ANOVA). A probability value of *p* < 0.05 was taken to be statistically significant.

## Results

### ApoE Deficiency Resulted in Atherosclerosis With a Significant Increase in Serum Ferritin and Aortic Iron and Ferritin in Mice

**FIGURE 1:**
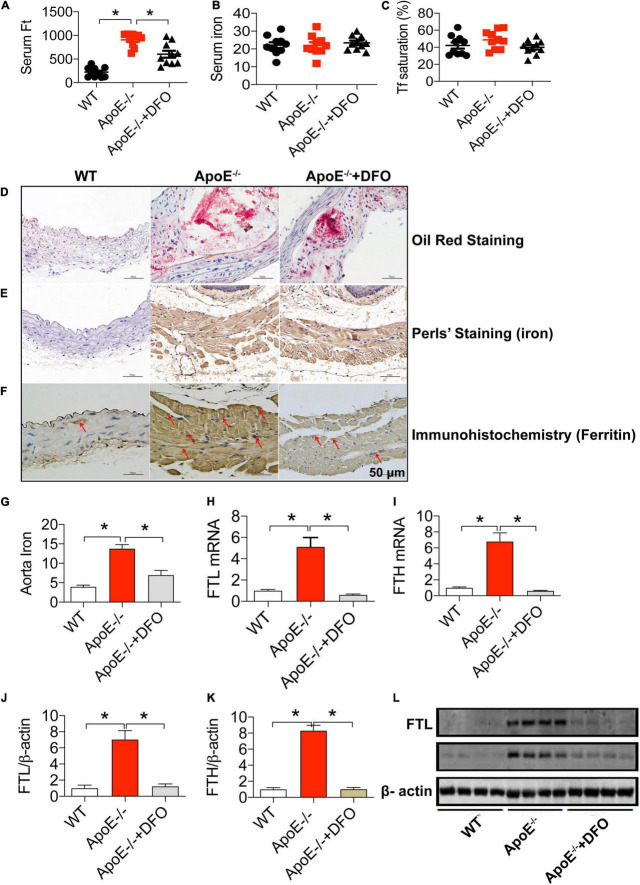
ApoE deficiency resulted in atherosclerosis with a significant increase in serum ferritin and aortic iron and ferritin in mice. The contents of ferritin (ng/mL) **(A)**, iron (μmol/L) **(B)** and Tf saturation (%) **(C)** in the serum, and Oil Red O staining **(D)**, DAB-enhanced Perls’ iron staining **(E)**, immunohistochemical analysis of FTL protein expression **(F)**, content of iron (mg/g wet weight) **(G)**, expression of FTL mRNA **(H)**, FTH mRNA **(I)**, FTL protein **(J,L)** and FTH protein **(K,L)** in the aortic tissues were determined or performed by the procedures as described in section “Materials and Methods” in WT (*n* = 10; WB: *n* = 4), ApoE^– /–^ (*n* = 10; WB: *n* = 4) and ApoE^– /–^ + DFO (*n* = 10; WB: *n* = 4) mice, all aged 28 week-old. The mice in ApoE^– /–^ + DFO group received daily i.p. injections of 100 mg/kg body weight (b.w.) of DFO in Hanks’ balanced salt solution (HBSS) for 14 days. Scale bar in panels **(D–F)** = 50 μm. Data are presented as the mean ± SEM. *p < 0.05.

We first investigated the effects of ApoE deficiency on the contents of iron and ferritin in the serum and the aortic tissues of mice in the presence or absence of DFO. As expected, ApoE deficiency induced atherosclerosis in aorta as reflected by Oil Red O staining (Figure 1D). Simultaneously, serum ferritin level (Figure 1A) was found to be significantly higher in ApoE–/– mice as compared to the WT mice. Also, the levels of iron (Figure 1G), FTL and FTH mRNAs (Figures 1H,I) and proteins (Figures 1J–L) in the aortic tissues in of ApoE–/– mice all were significantly higher than those in WT mice. The significantly increased iron and ferritin in the aorta were also confirmed by DAB-Enhanced Perls Staining (Figure 1E) and immunohistochemistry analysis (Figure 1F), indicating that iron accumulated in atherosclerotic vascular plaque in ApoE–/– mice. There were no differences in serum iron (Figure 1B) and Tf saturation (Figure 1C) between ApoE–/– and WT mice. The treatment with DFO was demonstrated to attenuate atherosclerosis (Figure 1D) with a significant reduction in ferritin in the serum (Figure 1A), iron (Figure 1G), FTL and FTH mRNAs (Figures 1H,I) and proteins (Figures 1C,J–L) in the aortic tissues in ApoE–/– mice. All of these measurements in ApoE–/– mice treated with DFO were significantly lower than those in ApoE-/- mice.

### Increased Expression of Transferrin Receptor 1 in the Aortic Tissues of ApoE Deficient Mice

**FIGURE 2:**
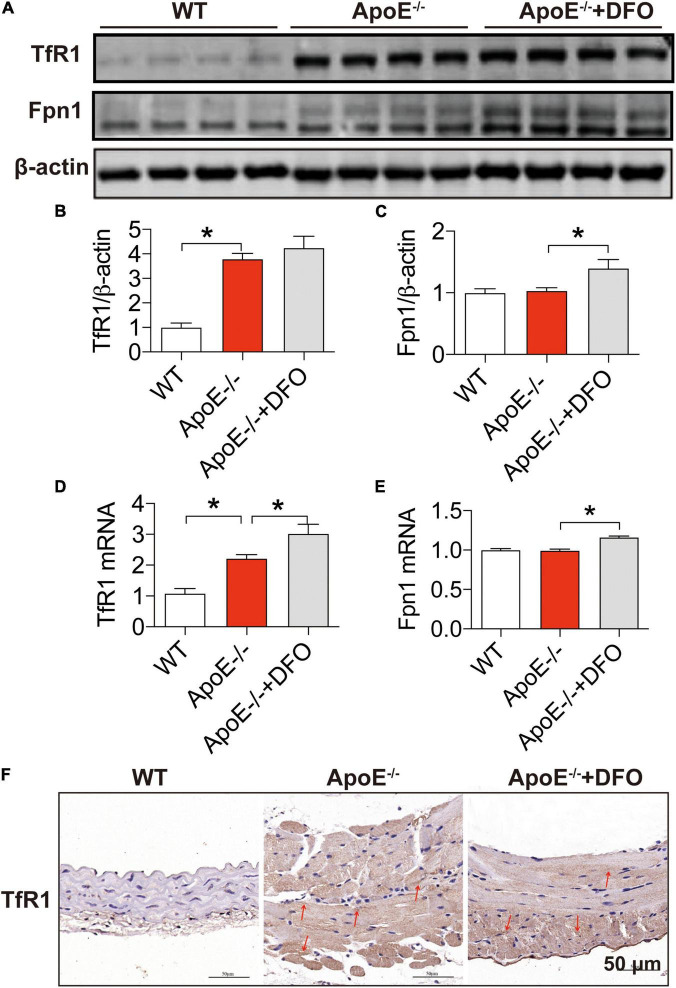
Increased expression of transferrin receptor 1 in the aortic tissues of ApoE deficient mice. The expression of TfR1 and Fpn1 proteins [TfR1, **(A,B)**; Fpn1, **(A,C)**] and mRNAs [TfR1, **(D)**; Fpn1, **(E)**] in the aortic tissues were, respectively, determined by Western blot and RT-PCR analysis as described in section “Materials and Methods” in WT (*n* = 8; WB: *n* = 4), ApoE^– /–^ (*n* = 8; WB: *n* = 4) and ApoE^– /–^ + DFO (*n* = 8; WB: *n* = 4) mice, all aged 28 week-olds. Immunohistochemical examination of TfR1 protein expression in the aortic tissues **(F)** was also conducted in the three groups of mice. The mice in ApoE^– /–^ + DFO group received daily i.p. injections of 100 mg/kg body weight (b.w.) of DFO in HBSS for 14 days. Scale bar in panel **(F)** = 50 μm. Data are presented as the mean ± SEM. *p < 0.05.

In most types of cells, iron balance is mainly dependent on the expression of two key cell-iron transporters, TfR1 (importer) (39, 40) and Fpn1 (exporter) (40). To find out the reason for the increased iron in aortic tissues, we then examined the expression of these two transporters in aortic tissues. Western blot and RT-PCR analysis demonstrated that ApoE deficiency induced a significant increase in the expression of TfR1 protein (Figures 2A,B) and mRNA (Figure 2D), but had no effect on that of Fpn1 protein (Figures 2A,C) and mRNA (Figure 2E) in aortic tissues of mice. The significantly increased expression of TfR1 protein in aortic tissues in ApoE deficient mice was also confirmed by immunohistochemistry (Figure 2F). These findings imply that ApoE deficiency could up-regulate TfR1 expression at post-transcriptional level and then cell iron uptake, which might be associated with the increased iron content in the aortic tissues of ApoE–/– mice. The treatment with DFO was found to increase the expression of TfR1 mRNA (Figure 2D), Fpn1 mNRA (Figure 2C) and protein (Figure 2E), indicating an increased activity of cell iron transportation in aortic tissues of ApoE–/– mice.

### ApoE Deficiency Up-Regulated IRP1 and IRP2 Expression but Not Hepcidin in the Aortic Tissues

**FIGURE 3:**
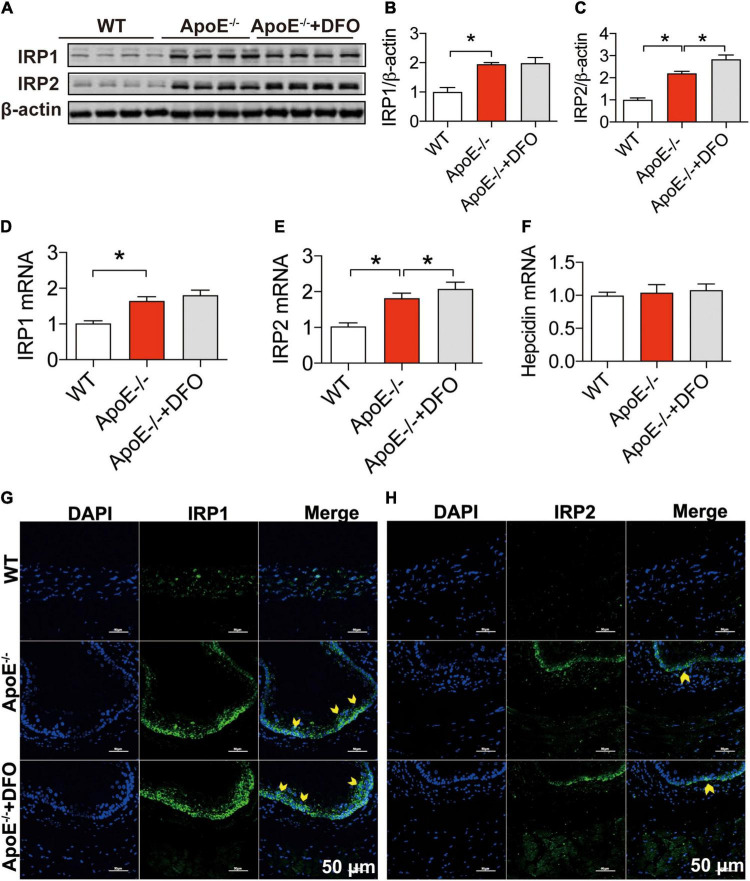
ApoE deficiency up-regulated IRP1 and IRP2 expression but not hepcidin in the aortic tissues. The expression of IRP1 and IRP2 proteins [IRP1, **(A,B)**; IRP2, **(A,C)**], mRNAs [IRP1, **(D)**; IRP2, **(E)**] and hepcidin mRNA **(F)** in the aortic tissues were, respectively, determined by Western blot and RT-PCR analysis as described in section “Materials and Methods” in WT (*n* = 8; WB: *n* = 3), ApoE^– /–^ (*n* = 8; WB: *n* = 3) and ApoE^– /–^ + DFO (*n* = 8; WB: *n* = 3) mice, all aged 28 week-old. Immunofluorescence analysis of IRPs protein expression in the aortic tissue **(G,H)** was also conducted in the three groups of mice. The mice in ApoE^– /–^ + DFO group received daily i.p. injections of 100 mg/kg body weight (b.w.) of DFO in HBSS for 14 days. Scale bar in panel **(H)** = 50 μm. Data are presented as the mean ± SEM. *p < 0.05.

To answer the question of how ApoE deficiency up-regulates TfR1 expression, we next investigated the effects of ApoE deficiency on the expression of IRP1, IRP2 and hepcidin in aortic tissues of the mice because the expression of TfR1 and Fpn1 is regulated not only by IRPs at the cellular level but also by hepcidin systemically (41, 42). Western blot and RT-PCR analysis demonstrated that ApoE deficiency could lead to a significant increase in the expression of IRP1 and IRP2 proteins (Figures 3A–C) and mRNAs (Figures 3D,E), but had no effect on that of hepcidin mRNA (Figure 3F) in aortic tissues of mice. The significantly increased expression of IRPs protein was also confirmed by immunofluorescence analysis (Figures 3G,H). The results suggested that the up-regulation of TfR1 induced by ApoE deficiency may be mediated by the increased expression of IRPs at the cellular level rather than hepcidin at systemic level. DFO treatment had no significant effects on expression of IRP1 protein (Figures 3A,B,G) and mRNA (Figures 3D,E) and IRP2 Protein (Figures 3A,C,H) and mRNA (Figure 3E), as well as hepcidin mRNA (Figure 3F) in the aortic tissues of ApoE–/– mice, although IRP2 Protein (Figures 3A,C,H) and mRNA (Figure 3E) expression was higher in ApoE–/– + DFO than ApoE–/– mice.

### ApoE Deficiency Up-Regulated HO1 Expression in the Aortic Tissues

**FIGURE 4:**
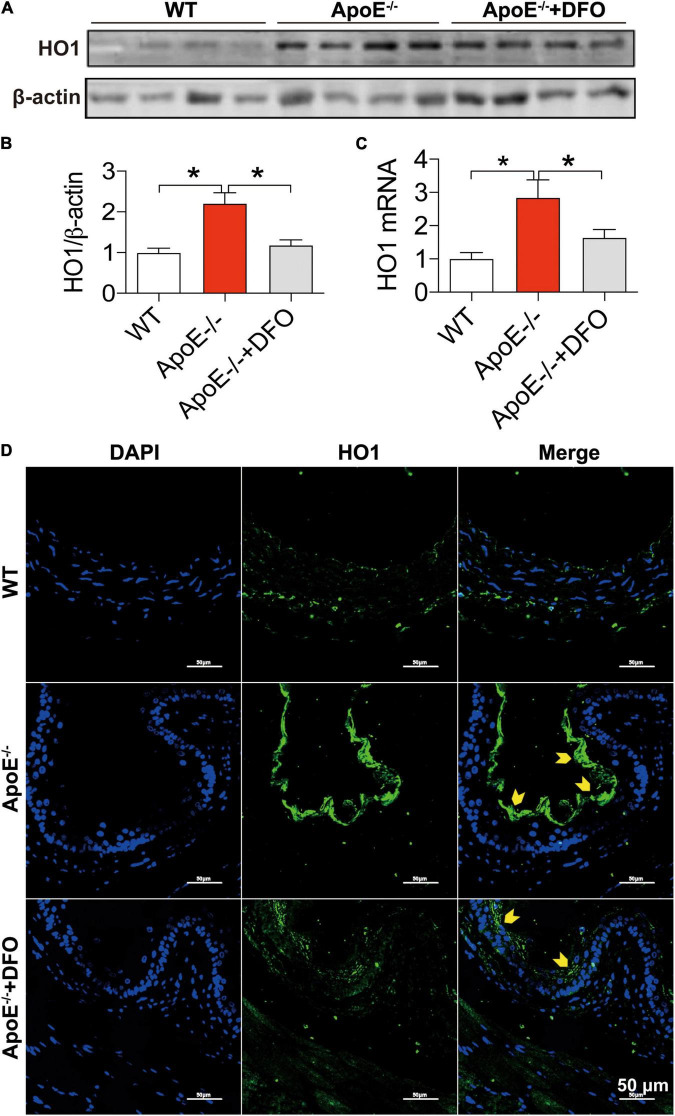
ApoE deficiency up-regulated HO1 expression in the aortic tissues. The expression of HO1 protein and mRNA in the aortic tissues were, respectively, determined by Western blot **(A,B)**, RT-PCR **(C)** or/and immunofluorescence **(D)** analysis as described in section “Materials and Methods” in WT (*n* = 8; WB: *n* = 4), ApoE^– /–^ (*n* = 8; WB: *n* = 4) and ApoE^– /–^ + DFO (*n* = 8; WB: *n* = 4) mice, all aged 28 week-old. The mice in ApoE^– /–^ + DFO group received daily i.p. injections of 100 mg/kg body weight (b.w.) of DFO in HBSS for 14 days. Scale bar in panel **(D)** = 50 μm. Data are presented as the mean ± SEM. *p < 0.05.

In addition to the cell-iron importer TfR1 and exporter Fpn1, HO1 is a critical contributor to cell-iron homeostasis by catabolizing heme and then releasing free iron. We therefore examined the changes in the expression of HO1 protein and mRNA in aortic tissues of WT and ApoE–/– mice. The levels of HO1 protein (Figures 4A,B,D) and mRNA (Figure 4C) in the aortic tissues of ApoE–/– mice were found to be significantly higher than those in WT mice, as demonstrated by Western blot (Figures 4A,B), RT-PCR (Figure 4C) and immunofluorescence analysis (Figure 4D). Treatment of ApoE–/– mice with DFO down-regulated significantly HO1 Protein (Figures 4A,B,D) and mRNA expression (Figure 4D).

### ApoE Deficiency Up-Regulated the Expression of ICAM1, VCAM1, LOX-1, CD36, Gpx4, TNFα, IL-1β, and IL-6 in the Aortic Tissues of Mice

**FIGURE 5:**
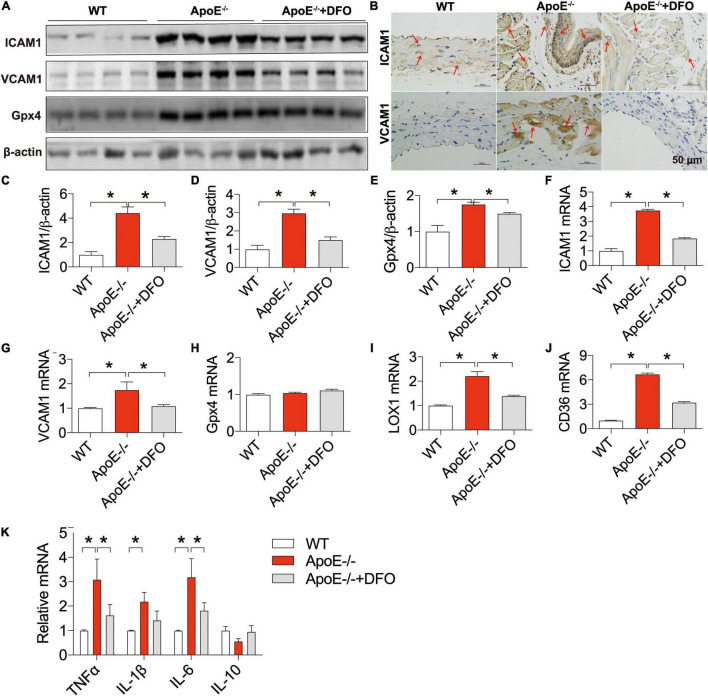
ApoE deficiency up-regulated the expression of ICAM1, VCAM1, LOX-1, CD36, Gpx4, TNFα, IL-1β, and IL-6 in the aortic tissues of mice. The expression of ICAM1, VCAM1 and Gpx4 proteins in the aortic tissues were measured by Western blot [ICAM1, **(A,C)**; VCAM1, **(A,D)**; Gpx4, **(A,E)**] or/and immunohistochemistry [ICAM1, **(B)**] analysis, and ICAM1 **(F)**, VCAM1 **(G)**, Gpx4 **(H)**, LOX-1 **(I)**, CD36 **(J)**, and inflammatory cytokines TNFα, IL-1β, IL-6 and IL-10 **(K)** mRNAs by RT-PCR analysis as described in section “Materials and Methods” in WT (*n* = 6; WB: *n* = 4), ApoE^– /–^ (*n* = 6; WB: *n* = 4) and ApoE^– /–^ + DFO (*n* = 6; WB: *n* = 4) mice, all aged 28 week-old. The mice in ApoE^– /–^ + DFO group received daily i.p. injections of 100 mg/kg body weight (b.w.) of DFO in HBSS for 14 days. Scale bar in panel **(B)** = 50 μm. Data are presented as the mean ± SEM. *p < 0.05.

We also examined the changes in the expression of ICAM1 and VCAM1 (cell adhesion molecules), LOX-1 (the main ox-LDL receptor of endothelial cells), CD36 and Gpx4 (a phospholipid hydroperoxidase that protects cells against membrane lipid peroxidation) (43) in the aortic tissues of ApoE deficient mice in the presence or absence of DFO. Western blot, RT-PCR and/or immunohistochemistry analysis showed that the expression of ICAM1 protein (Figures 5A–C) and mRNA (Figure 5F), VCAM1 protein (Figures 5A,B,D) and mRNA (Figure 5G), Gpx4 protein (Figures 5A,E), LOX-1 mRNA (Figure 5I), and CD36 mRNA (Figure 5J) in the aortic tissues was significantly higher in ApoE–/– mice than in WT mice except for Gpx4 mRNA (Figure 5H). These results indicated that ApoE deficiency up-regulated the expression of ICAM1, VCAM1, LOX-1 and CD36 at post-transcriptional level and Gpx4 at translational level in the aortic tissues of mice. The expression of these molecules was significantly lower in ApoE deficient mice treated with DFO than in ApoE deficiency mice.

The expression of ICAM1, VCAM1, LOX-1 and CD36 can be up-regulated following exposure to pro-inflammatory and pro-atherogenic stimuli (44–46). Thus, we also examined the expression of inflammatory cytokines TNFα, IL-1β, IL-6 and IL-10. It was found that the expression of TNFα, IL-1β and IL-6 mRNAs in the aortic tissues was significantly higher in ApoE–/– mice than in WT mice except for IL-10 mRNA which was slightly lower in ApoE–/– mice (Figure 5K). Treatment with DFO led to a reduction in the expression of TNFα, IL-1β and IL-6 mRNAs and had no effect on IL-10 in aorta of ApoE–/– mice (Figure 5K), implying that DFO has the ability to attenuate TNFα, IL-1β and IL-6 production and then inhibit the expression of ICAM1, VCAM1, LOX-1 and CD36 in ApoE–/– mice.

### Increased Reactive Oxygen Species Production, NF-κB Phosphorylation and Reduced Superoxide Dismutase and GSH-PX Contents in ApoE Deficient Mice

**FIGURE 6:**
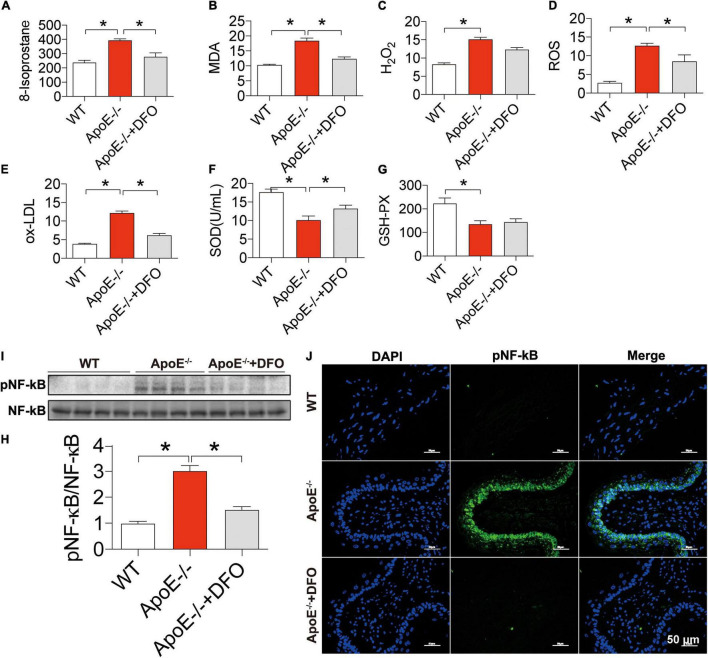
Increased production of reactive oxygen species, NF-κ B phosphorylation and reduced SOD and GSH-PX contents in ApoE deficient mice. The contents of 8-isoprostane (pg/mL) **(A)**, MDA (nmol/mL) **(B)**, H_2_O_2_ (nmol/mL) **(C)**, ROS (Fold of the Control) **(D)** and ox-LDL (μg/mL) **(E)**, SOD (U/mL) **(F)** and GSH-PX **(G)** in the blood, and NF-κB phosphorylation [**(H,I)** NF-kB family contains five members: p50 (NF-kB1), p52 (NF-kB2), p65 (RelA), c-Rel, and RelB, and panel **(J)**] in the aortic tissues were determined by the procedures as described in section “Materials and Methods” in WT (*n* = 8), ApoE^– /–^ (*n* = 8) and ApoE^– /–^ + DFO (*n* = 8) mice, all aged 28 week-old. The mice in ApoE^– /–^ + DFO group received daily i.p. injections of 100 mg/kg body weight (b.w.) of DFO in HBSS for 14 days. Scale bar in panel **(J)** = 50 μm. Data are presented as the mean ± SEM. *p < 0.05.

Finally, we examined the effects of ApoE deficiency on the production of ROS and the contents of SOD and GSH-PX in the blood of mice. 8-isoprostane (Figure 6A), MDA (Figure 6B), H2O2 (Figure 6C), ROS (Figure 6D), and ox-LDL (Figure 6E) levels were significantly higher in ApoE–/– mice than in WT mice and DFO-treated ApoE–/– mice, indicating that ApoE deficiency could promote oxidative stress, while DFO can suppress this promotion. On the other hand, ApoE deficiency was found to induce a significant reduction in the contents of SOD (Figure 6F) and GSH-PX (Figure 6G) in the blood of mice, while DFO increased SOD, but had no effect on GSH-PX level in ApoE–/– mice. In addition, western blot and immunofluorescence results showed that ApoE deficiency induced a significant increase in NF-κB/pNF-κB in the aortic tissues, while DFO treatment could effectively attenuate this effect (Figures 6H–J).

## Discussion

In spite of years’ studies, it is still controversial and an unsolved issue of whether iron is involved in the development of atherosclerosis. Also, it is still unknown whether iron deposition in the aortic tissues is a cause or simply a downstream consequence of atheroma formation (13). One of the key points to answering these questions is to know the mechanisms of how ApoE deficiency causes iron deposition in the aortic tissues. In the present study, we demonstrated that ApoE deficiency induces iron-overload as well as atherosclerosis in the aortic tissues. The development of atherosclerosis is confirmed by Oil Red O staining and the significant increase in the levels of iron and ferritin was demonstrated by direct measurement of total iron content and also by western blot and RT-PCR analysis in the aortic tissues of ApoE–/– mice. The increased iron and ferritin in the aortic tissues were also confirmed by DAB-Enhanced Perls Staining and immunohistochemistry analysis. Also, we showed that ApoE deficiency induced an increase in iron content and ferritin expression in the aortic tissues and the extent of atherosclerotic severity both could be significantly abolished by DFO treatment.

**FIGURE 7:**
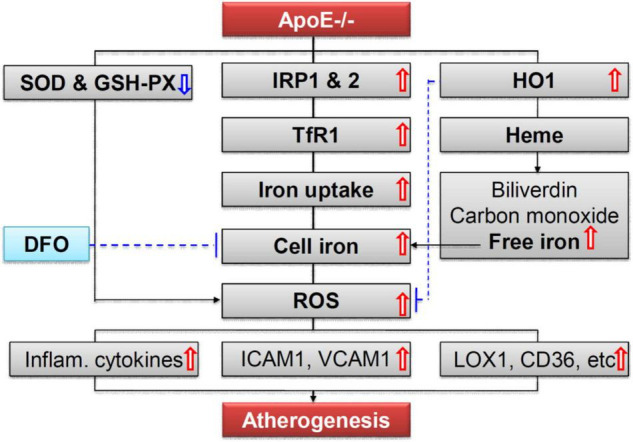
A hypothetical scheme for the role of iron in ApoE deficiency-induced atherosclerosis. The increased iron accumulation in the aortic tissues (AET) may be mainly due to increased cell-iron uptake *via* IRP/TfR1 pathway. Increased ROS production, induced mainly by increased iron and possibly also partly by reduction in SOD and GSH-PX enzymes, plays an important role in endothelial activation and consequent monocyte recruitment to the arterial intimae, *via* up-regulation of the expression of cellular adhesion molecules ICAM1 and VCAM1, main ox-LDL receptor of endothelial cells LOX-1, important receptor for oxidized lipoproteins CD36, NF-κB phosphorylation, and pro-inflammatory cytokines TNFα, IL-1β, and IL-6, in the aortic tissues of mice.

More importantly, we demonstrated that ApoE deficiency resulted in a significant increase in the expression of TfR1 (a key protein for cell-iron uptake), but not Fpn1 (the only-known cell-iron exporter), and IRP1 and IRP2 but not hepcidin, in the aortic tissues of mice, by western blot and RT-PCR and/or immunofluorescence analysis. The findings indicated that increased iron and ferritin in the aortic tissues is due to the increased iron uptake by, rather than the reduced release from the aortic cells or tissues. Because cell or tissue iron levels mainly depend on expression of TfR1 and Fpn1, while the expression of TfR1 and Fpn1 is regulated cellularly by IRPs and systemically by hepcidin (41, 47, 48), the increased iron uptake therefore is induced by the increased expression of TfR1 resulting from the increased IRPs at the cellular level, rather than by hepcidin at systemic level in ApoE deficient mice. These findings imply that the increased cell-iron uptake *via* IRP/TfR1 pathway is one of the reasons for the increased iron in the aortic tissues of ApoE–/– mice (Figure 7) although the mechanisms of how ApoE deficiency up-regulates IRPs’ expression have yet to be clarified. These findings also provide solid evidence for a key role of iron in the development of atherosclerosis and support the possibility that the increased iron in the aortic tissues may be one of the causes rather than a downstream consequence of the atheroma formation in ApoE deficient mice. In addition,

We also found that ApoE deficiency induced a significant increase in the production of ROS which was demonstrated by the up-regulation of 8-isoprostane, MDA, H2O2 and ox-LDL and a reduction in the contents of SOD and GSH-PX enzyme in ApoE–/– mice. Also, these changes induced by ApoE deficiency could also be reversed by DFO treatment. These findings imply that the increased production of ROS is induced by the increased iron and might also be partly associated with the reduction in SOD and GSH-PX expression. In addition, ApoE deficiency was found to up-regulate the expression of cellular adhesion molecules ICAM1 and VCAM1, the main ox-LDL receptor of endothelial cells LOX-1, an important receptor for oxidized lipoproteins CD36, NF-κB phosphorylation, and pro-inflammatory cytokines TNFα, IL-1β, and IL-6 in the aortic tissues of mice (Figure 7). Also, these changes were found to be abolished by DFO treatment. These data are consistent with those reported by others (7, 49–53) and provide further evidence for the notion that iron mediated ROS production, by stimulating redox-sensitive cell signaling pathways, plays an important role in endothelial activation which is characterized by up-regulation of cellular adhesion molecules and pro-inflammatory chemokines and cytokines, and consequent monocyte recruitment to the arterial intimae (5, 7, 49–51, 54).

Gpx4 is a phospholipid hydroperoxidase that protects cells against membrane lipid peroxidation (43) and is also a key inhibitor of ferroptosis, a recently recognized form of regulated cell death (55–57). In the present study, ApoE deficiency induced a significant increase in the expression of Gpx4 protein, but not mRNA in the aortic tissues, indicating an up-regulation of Gpx4 by ApoE deficiency at a translational level in the aortic tissues of mice. A significant reduction in Gpx4 protein in DFO-treated ApoE–/– mice implies that the regulation of Gpx4 expression by ApoE deficiency is an iron-associated process. The increased expression of Gpx4 in ApoE deficient-mice supports the idea that ApoE deficiency could induce a protective response against membrane lipid peroxidation and also suggested that ferroptosis, an iron-dependent process of cell death (55–57), might not be associated with the development of atherosclerotic lesion although the latter is iron-associated.

A persistent criticism of the iron hypothesis or the role of iron in atherosclerosis has been that atherosclerosis may not be a prominent feature of hereditary hemochromatosis (HH), and the essence of this criticism is that iron cannot be a significant factor in atherosclerosis in those unaffected by inherited iron overload unless an increase in atherosclerosis is observed in HH (58). At first glance, this criticism may sound reasonable, but on closer examination, we can find that it does not hold water. Based on current knowledge, tissues or organs that have excess iron accumulation are different in different hereditary iron overload disorders such as HH (4, 59) and hereditary aceruloplasminemia (HA) (60, 61). Also, it is well-known that not all tissues, organs and cells are overloaded with iron in these disorders including HH and HA. The key question is whether there is excess iron accumulation in the aortic tissues, which is the location of atherosclerosis, in these hereditary iron overload disorders.

In the case of HH, the key is whether sequence variations in HFE gene, especially the most common variations of C282Y and H63D, can initiate iron-overload in the aortic tissues, and if not, how an increase in atherosclerosis could be observed in HH in spite of the existence of excess iron accumulation in multiple tissues including the heart. It is reasonable to believe that “an increase in atherosclerosis” should not have been observed in HH if there is not excess iron accumulation in the aortic tissues or if sequence variations in HFE gene cannot initiate excess iron accumulation in the aortic tissues. Neurodegeneration, same as atherosclerosis, is also not a prominent feature of HH, however, few would agree with the claim that iron cannot be a significant factor in neurodegeneration unless an increase in neurodegeneration is observed in HH. Neurodegeneration is not a prominent feature of HH because iron homeostasis in the brain is not significantly affected in HH. For the same reason, atherosclerosis is not a prominent feature of HH also because iron homeostasis in the aortic tissues is not significantly affected in HH.

In fact, any assessments of the effect of “systemic/body iron overload” or “increased body iron” (if not accompanied by the increased iron in the aortic tissues) on atherosclerosis will not inform us about whether iron promotes atherosclerosis. The “systemic/body iron overload” or “increased body iron” does not necessarily lead to the “increased iron content in the aortic tissues.” Only the investigations on the effect of “increased iron content in the aortic tissues” rather than “systemic/body iron overload” or “increased body iron” on atherosclerosis can contribute to our understanding of whether iron promotes atherosclerosis. It is not surprising that the results obtained from studies on the effects of “increased iron content in the aortic tissues on atherosclerosis” were inconsistent with those on the effects of “increased body (liver or others) iron on atherosclerosis.” Also, it may be expected that all investigations on the effect of “increased iron content in the aortic tissues” on atherosclerosis will generate consistent rather than inconsistent results. In short, “the excess iron accumulation in multiple tissues including the heart in HH” may not be relevant to “the role of iron in the development of atherosclerosis.”

The fact that atherosclerosis is not a prominent feature of HH in spite of the existence of excess iron accumulation in multiple tissues very strongly supports that body iron overload, if not accompanied by the “increased iron content in the aortic tissues,” is not a significant factor in atherosclerosis, but the role of iron in ApoE deficiency-induced atherosclerosis cannot be completely denied because iron contents are increased in the aortic tissues of ApoE–/– mice. In a recent study, we demonstrated that ApoE deficiency could induce a progressive increase in iron contents of the liver and spleen with age and replenishment of absent ApoE can reverse the iron-related phenotype in ApoE–/– mice, indicating that ApoE may be essential for physiological iron metabolism (21). Further studies on the physiological role of ApoE in body iron homeostasis may be able to provide key insights into understanding whether iron can initiate atherosclerosis in ApoE deficient mice.

A number of studies have confirmed that chronic iron overload by injection with iron-dextran (62) or high iron diet (63) intensifies the atherosclerotic process with an increase in iron deposition in the aorta, while iron depletion by iron chelation (7, 64–66) and dietary iron restriction (20, 63, 66) attenuates the atherosclerosis progression with a reduction in iron deposit in the aortic tissues or macrophage iron content in plaques in ApoE–/– mice. However, conflicting results have also been reported, showing that high−iron diet or intravenous iron injection (65) is not atherogenic (67) and even decreases the atherosclerosis progression (68).

Kirk et al. (68) compared the effects of a standard diet (0.02% iron) and a 2% carbonyl iron diet on atherosclerosis in ApoE–/– mice and demonstrated that 2% iron diet did not exacerbate, but rather reduced the severity of atherosclerosis, although levels of ferritin and iron both were significantly higher in liver and serum from mice fed the 2% iron diet than those from controls. However, more importantly, they also showed that there are no differences in ferritin protein in aortic tissue between the 2% and 0.02% iron diet groups, indicating that 2% iron diet has no effect on iron content in aortic tissue under their experimental conditions. This should be one of the key causes of why 2% iron diet did not exacerbate the severity of atherosclerosis because the severity of atherosclerosis depends mainly on the iron contents in aortic tissue rather than in the liver and serum as we discussed in above. Their findings provide further support that only excess iron accumulation in the aortic tissues (the location of atherosclerosis), rather than elevated levels of iron in the liver and serum, is associated with the development of atherosclerosis.

It has also been reported that hepatic hepcidin expression was not increased at any stage of atherosclerosis progression in ApoE–/– or ApoE/ffe mice and that the atherosclerotic plaque size was not increased in mice with elevated macrophage iron by intravenous iron injection (weekly iron sucrose injections for 8 weeks,16 mg iron total) (67). It was therefore concluded that macrophage iron has no significant role in atherosclerosis progression in mice. However, macrophage iron loading induced by iron sucrose injections was confirmed by quantitating non-heme iron contents in the liver and spleen (67), rather than macrophage iron content in atherosclerotic plaques, which has been demonstrated to be a critical factor in progression of atherosclerosis (65). Therefore, their results strongly argue against any significant role of “macrophage iron in the liver and spleen,” rather than “macrophage iron in the atherosclerotic plaques,” in atherosclerosis progression in mice, and thus cannot disavow the role of increased iron in the aortic tissues in the development of atherosclerosis in ApoE deficient mice.

Hepcidin is the main regulator of body iron levels and its tissue distribution (69, 70). Iron is exported from the cells or tissues through Fpn1, the sole cellular iron efflux channel and the hepcidin receptor. However, hepcidin/Fpn1 axis may not play a major role in atherosclerosis progression in ApoE–/– mice. In the present study, we found that ApoE deficiency has no significant effect on the expression of hepcidin (and also Fpn1) in ApoE–/– mice, being consistent with what was reported by Kautz et al. (67), namely hepcidin expression was not increased at any stage of atherosclerosis progression in ApoE–/– mice. Therefore, un-charged expression of hepcidin at any stage of atherosclerosis progression in ApoE–/– mice (67) cannot be considered as evidence denying the role of increased iron contents in the aortic tissues in ApoE deficiency-induced atherosclerosis in mice.

In conclusion, our findings demonstrate that ApoE deficiency could up-regulate the expression of IRPs (but not hepcidin) and TfR1 (and not Fpn1), leading to an increase in iron uptake by the aortic tissues and cells. The increased iron accumulation in the aortic tissues may be mainly due to increased cell-iron uptake *via* IRP/TfR1 pathway. These findings also support the idea that iron mediated ROS production plays an important role in endothelial activation and consequent monocyte recruitment to the arterial intimae. Based on our findings and also the discussion and analysis of the controversial results regarding whether iron is involved in atherosclerosis as reported by other groups, we concluded that ApoE deficiency-induced atherosclerosis is at least partly mediated by the increased iron in the aortic tissues.

## Data Availability Statement

All data and materials generated or analyzed during this study are included in this article.

## Ethics Statement

The animal study was reviewed and approved by the Animal Management Rules of the Ministry of Health of China; Animal Ethics Committees of Nantong University (NDFC31271132) and The Chinese University of Hong Kong (GRF14111815). All applicable international, national, and/or institutional guidelines for the care and use of animals were followed.

## Author Contributions

YK and Z-MQ conceived, organized, and supervised the study and prepared, wrote, and revised the manuscript. JM, H-MM, M-QS, Y-XB, and YL carried out the animal and cell-experiments and collected data. JM and YW contributed to the analysis of data and generated figures. All authors contributed to the article and approved the submitted version.

## Conflict of Interest

The authors declare that the research was conducted in the absence of any commercial or financial relationships that could be construed as a potential conflict of interest.

## Publisher’s Note

All claims expressed in this article are solely those of the authors and do not necessarily represent those of their affiliated organizations, or those of the publisher, the editors and the reviewers. Any product that may be evaluated in this article, or claim that may be made by its manufacturer, is not guaranteed or endorsed by the publisher.

## Abbreviations

ApoE: Apolipoprotein E
ApoE^–/–^: Apolipoprotein E-deficiency
BSA: Bovine serum albumin
CD36: An important receptor for oxidized lipoproteins
DAB: 3,3 ′ -diaminobenzidine
DAPI: 4 ′ ,6-diamidino-2-phenylindole
DFO: Deferoxamine
FBS: Fetal bovine serum
Fpn1: Ferroportin
FTH: Ferritin heavy chain
FTL: Ferritin light chain
Gpx4: Glutathione peroxidase 4
GSH-PX: Glutathione peroxidase
HBSS: Hanks’ balanced salt solution
HO-1: Heme oxygenase 1
HRP: Horseradish peroxidase
HUVECs: Human umbilical vein endothelial cells
ICAM1: Intercellular adhesion molecule-1
IL: Interleukin
IRP1: Iron regulatory protein 1
IRP2: Iron regulatory protein 2
LDL: Low density lipoprotein
LOX-1: Lectin-like Ox-LDL receptor 1
MDA: Malondialdehyde
NF-kB: Nuclear Factor-kB
ox-LDL: Oxidized-low density lipoprotein
PBS: Phosphate buffer saline
ROS: Reactive oxygen species
SOD: Superoxide Dismutase
TfR1: Transferrin receptor 1
TNF-α: Tumor necrosis factor- α
UIBC: Unsaturated iron binding capacity
VCAM1: Vascular cell adhesion molecule-1
WT: Wild-type.
