# Supplementary material for: The Role of Iron in Atherosclerosis in Apolipoprotein E Deficient Mice
Source: Front Cardiovasc Med. 2022 May 20;9:857933. doi: 10.3389/fcvm.2022.857933 (PMC9163807; doi:10.3389/fcvm.2022.857933)
Supplement: Supplementary file 1 [file Table_1.pdf]

Table 1. Antibodies

Antibody	Vendor	Catalogue number
Anti-Fpn1	Novus Biologicals	NBP1-21502
Anti-FTH	Bioworld Technology	BS6175
Anti-FTL	Proteintech	10727-1-AP
Anti-Gpx4	Abcam	ab125066
Anti-HO-1	Abcam	ab68477
Anti-ICAM1	Abcam	ab179707
Anti-IRP1	Abcam	ab126595
Anti-IRP2	Abcam	ab181153
Anti-NF-kB p65	Cell signaling	8242
Anti-p-NF-kB p65	Cell signaling	3033
Anti-p-STAT3	Cell signaling	9131
Anti-VCAM1	Abcam	ab134047
Anti-TfR1	Life Technologies	13-6800
beta-actin	Sigma	A2228
