# Supplementary figures and images for: The Role of Iron in Atherosclerosis in Apolipoprotein E Deficient Mice
Source: Front Cardiovasc Med. 2022 May 20;9:857933. doi: 10.3389/fcvm.2022.857933 (PMC9163807; doi:10.3389/fcvm.2022.857933)

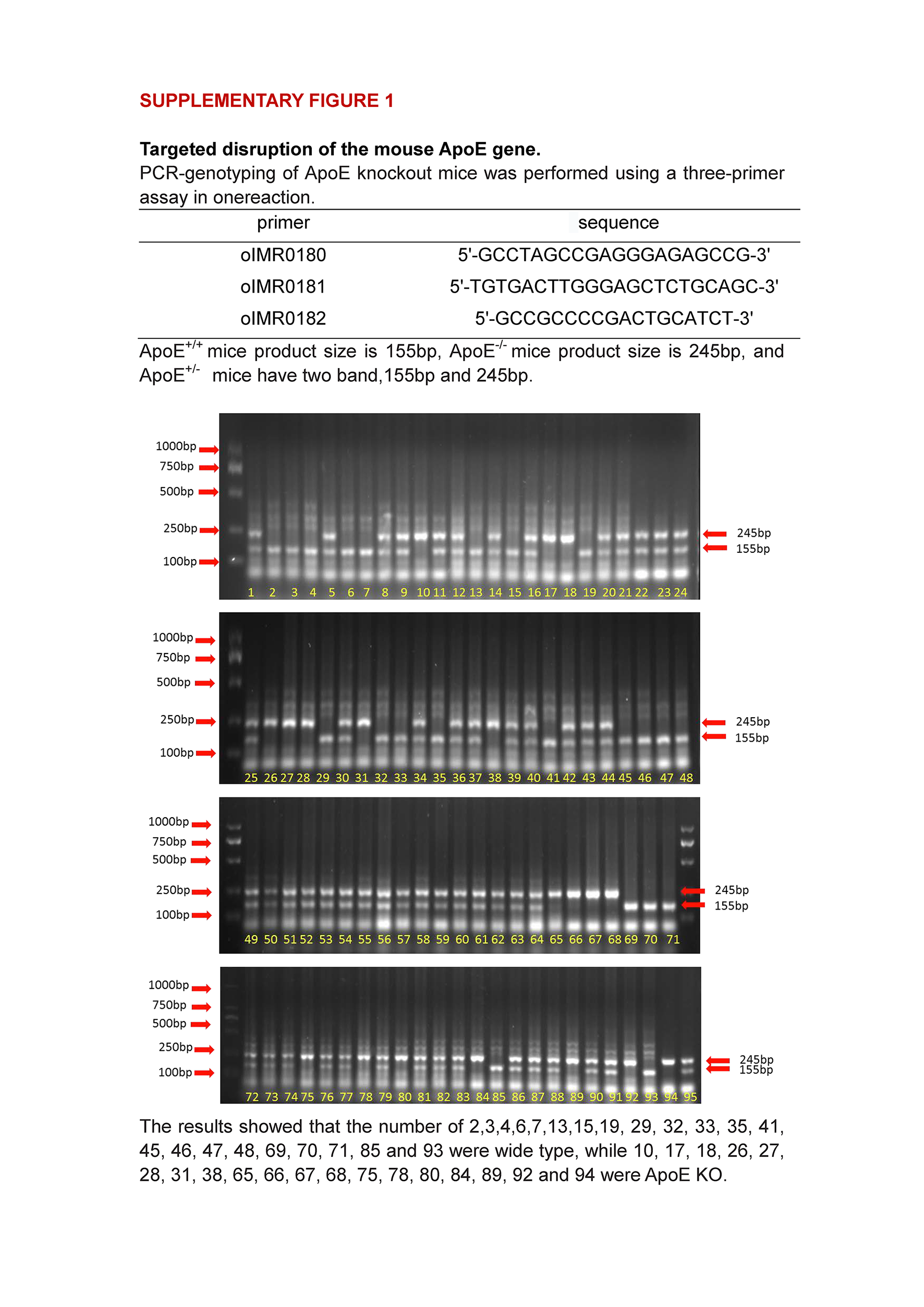

Supplement: Supplementary file 2 [file Image_1.TIFF]
